# The Clinical Significance of Aspergillus Detected in Lower-Respiratory-Tract Samples of Critically Ill COVID-19-Positive Patients

**DOI:** 10.3390/arm91050027

**Published:** 2023-09-02

**Authors:** Heba Osman, Asra N. Shaik, Paul L. Nguyen, Zachary Cantor, Mirna Kaafarani, Ayman O. Soubani

**Affiliations:** 1 Department of Medicine and Pediatrics, Wayne State University School of Medicine, Detroit, MI 48201, USA; 2 Department of Medicine, Wayne State University School of Medicine, Detroit, MI 48201, USA; 3 Division of Pulmonary, Critical Care and Sleep Medicine, Wayne State University School of Medicine, 3990 John R-3 Hudson, Detroit, MI 48201, USA

**Keywords:** *Aspergillus*, COVID-19, SARS-CoV-2, superimposed infection, invasive pulmonary aspergillosis

## Abstract

**Highlights:**

**What are the main findings?**

- In this retrospective single-center cohort study, we characterized the incidence and outcomes of hospitalized COVID-19 patients with *Aspergillus* isolated from lower-respiratory-tract samples.
- We found that critically-ill patients with COVID-19 were at increased risk for co-infection with *Aspergillus*, and patients with isolated *Aspergillus* suffered a high mortality rate.

**What are the implications of the main findings?**

- Our study findings support testing for the presence of *Aspergillus* in lower-respiratory secretions and appropriate additional studies for critically ill patients with COVID-19.
- Early identification of patients with COVID-19-associated invasive pulmonary aspergillosis is critical to initiate prompt anti-fungal treatment.

**Abstract:**

**Objective**: Critically ill patients with acute respiratory distress syndrome (ARDS) due to viral infection are at risk for secondary complications, including invasive aspergillosis. Our study aimed to characterize the clinical significance and outcome of *Aspergillus* species isolated from lower-respiratory-tract samples of critically ill OVID-19 patients at a single center. **Design**: We conducted a retrospective cohort study to evaluate the characteristics of patients with COVID-19 and *aspergillus* isolated from the lower respiratory tract and to identify predictors of outcomes in this population. **Setting**: The setting was a single-center hospital system within the metropolitan Detroit region. **Results**: The prevalence of *Aspergillus* isolated in hospitalized COVID-19 patients was 1.18% (30/2461 patients), and it was 4.6% in critically ill ICU patients with COVID-19. Probable COVID-19-associated invasive pulmonary aspergillosis (CAPA) was found in 21 critically ill patients, and 9 cases were classified as colonization. The in-hospital mortality of critically ill patients with CAPA and those with *aspergillus* colonization were high but not significantly different (76% vs. 67%, *p* = 1.00). Furthermore, the in-hospital mortality for ICU patients with or without *Aspergillus* isolated was not significantly different 73.3% vs. 64.5%, respectively (OR 1.53, CI 0.64–4.06, *p* = 0.43). In patients in whom *Aspergillus* was isolated, antifungal therapy (*p* = 0.035, OR 12.3, CI 1.74–252); vasopressors (0.016, OR 10.6, CI 1.75–81.8); and a higher mSOFA score (*p* = 0.043, OR 1.29 CI 1.03–1.72) were associated with a worse outcome. In a multivariable model adjusting for other significant variables, FiO_2_ was the only variable associated with in-hospital mortality in patients in whom *Aspergillus* was isolated (OR 1.07, 95% CI 1.01–1.27). **Conclusions**: The isolation of *Aspergillus* from lower-respiratory-tract samples of critically ill patients with COVID-19 is associated with high mortality. It is important to have a low threshold for superimposed infections such as CAPA in critically ill patients with COVID-19.

## 1. Background

COVID-19 (Coronavirus 2019), the novel Coronavirus SARS-CoV-2, caused one of the deadliest pandemics in recent history [1]. Currently, there are substantial data regarding outcomes in patients with a COVID-19 infection, with mortality ranging from 9.06% to 15.65% [2]. However, data regarding hospital outcomes for patients with COVID-19 infection and co-existing conditions are limited. Furthermore, while bacterial or viral co-infections associated with COVID-19 have been reported, data on *Aspergillus* co-infection in patients with COVID-19, otherwise known as COVID-19-associated invasive pulmonary aspergillosis (CAPA), are scarce.

Invasive pulmonary aspergillosis (IPA) is a life-threatening disease and thought to usually occur in immunocompromised hosts [3,4]. It can lead to pulmonary involvement, most notably hypoxic respiratory failure secondary to pneumonia, requiring intubation and mechanical ventilation [5]. Additionally, IPA is associated with high mortality, presenting a fatality rate of 12.0–39% [6,7,8]. Factors driving the high fatality rate include difficulty diagnosing IPA given its non-specific symptoms and a lack of efficacious microbiological testing [9]. The incidence of CAPA is unclear due to varying definitions and diagnostic criteria among studies [10].

*Aspergillus* was detected in 2% of endotracheal aspirate cultures of mechanically ventilated ICU patients and likely represented colonization rather than active infection [11]. While the significance of *Aspergillus* isolated from respiratory cultures has been studied extensively in immunocompromised patients who develop IPA, the significance of *Aspergillus* isolated from respiratory cultures is not well-characterized in immunocompetent patients [12,13,14]. Therefore, the applicability of the European Organization for Research and Treatment of Cancer Mycoses Study Group (EORTC-MSG) consensus criteria used to define invasive aspergillosis in immunocompromised patients is heavily debated [15]. Another algorithm described by Blot et al. was developed to successfully differentiate the colonization of *Aspergillus* species from putative IPA for patients in ICUs on the basis of mycological criteria, combining cultures from respiratory specimens and galactomannan detection in the bronchoalveolar lavage (BAL) and serum [16]. The AspICU algorithm used clinical signs, less restrictive host factors, and an *Aspergillus*-positive culture from the respiratory tract to define putative aspergillosis. However, Blot et al. did not use galactomannan antigen detection in their definition [16]. The definition of invasive fungal diseases was again updated in 2019 by EORTC-MSG to include *Aspergillus* quantitative polymerase chain reaction (qPCR) and ß-D-glucan [15].

Viral respiratory infections, namely influenza, have long been associated as an independent risk factor for developing IPA [17]. Furthermore, data reveal an increased incidence between IPA and COVID-19 infection [13,14,15,16]. Machado et al. reported eight cases of CAPA, in 3.3% of 239 ICU patients, and five cases of *Aspergillus* colonization. Machado et al. found that CAPA primarily effected non-immunocompromised patients (75%) with severe ARDS receiving corticosteroids. CAPA-related mortality compared to colonized patient mortality was 100% vs. 40% (*p* = 0.04) [18]. The authors additionally reviewed 96 similar cases of CAPA and *Aspergillus* colonization from recent publications and estimated that the overall mortality was 56.3% [18]. The majority of studies suggest that proven or probable/putative CAPA mostly occurs in critically ill mechanically ventilated COVID-19 patients, with an incidence range 3–20% [19,20,21,22,23]. A recent prospective study of 108 patients found that probable CAPA was diagnosed in 30 (27.7%) patients after a median of 4 days from ICU admission, highlighting that CAPA may be more common than previously suggested [24].

Our objective here was to describe the significance and outcome of *Aspergillus* isolated from lower-respiratory-tract samples in hospitalized COVID-19 patients.

## 2. Methods

### 2.1. Study Design

We conducted a retrospective cohort study within a major tertiary medical center in Detroit. All research was conducted in accordance with the institutional review boards (IRB Protocol #20-04-2179). No consent was obtained for this study, as our analysis was on retrospective, de-identified data, for which our IRB rules waived the need for patient consent. No external funding was involved in conducting this study.

Thirty patients were initially identified as candidates for this study, as ascertained between 1 March 2020 and 1 May 2021. Patients were eligible for inclusion for the study if they (1) were positive for SARS-CoV-2 PCR, (2) had at least one lower-respiratory-tract sample positive for *Aspergillus* species, and (3) were at least 18 years of age (Supplemental Figure S1).

Every COVID-19-positive adult patient with *Aspergillus* spp. isolated from at least one respiratory sample was followed and classified as proven aspergillosis, probable aspergillosis, or colonization. Lower-respiratory-tract samples were collected by tracheal suctioning (6.7%) or with bronchoalveolar lavage (BAL) during bronchoscopy (93.3%). Electronic medical records were reviewed, including demographic, clinical, and radiological features; treatment; and outcomes.

### 2.2. Definition of Probable Invasive Pulmonary Aspergillosis and Aspergillus Colonization

Patients were classified as having CAPA according to the modified AspICU algorithm with the addition of fungal biomarkers [25]. For probable CAPA, patients met all four criteria:

1. *Aspergillus*-positive from lower-respiratory-tract culture sample.
2. Compatible signs and symptoms—one or more of the following: fever refractory to at least three days of appropriate antibiotic therapy, recrudescent fever after a period of defervescence, pleuritic chest pain, pleuritic rub, dyspnea, hemoptysis, and worsening respiratory insufficiency despite appropriate antibiotic therapy and ventilatory support (in line with the modified AspICU criteria).
3. Abnormal chest radiograph or computed tomography scan.
4. The presence of either:
  - One of the following host risk factors—neutropenia, underlying hematological or oncological malignancy, glucocorticoid treatment with prednisone equivalent > 20 mg/day, and congenital or acquired immunodeficiency.
  - A positive cytological smear showing branching hyphae. Galactomannan (GM) detection values in BAL or tracheal aspirate, or positive serum sample (>0.5) were added to this algorithm as a microbiological criterion (modified AspICU).

If a patient had an *Aspergillus*-positive culture from the lower-respiratory-tract sample but did not meet all other criteria, their case was classified as *Aspergillus* colonization.

### 2.3. COVID Dataset

The number of patients hospitalized in our main facilities with COVID-19 from 1 March 2020 to 1 May 2021 was obtained.

### 2.4. Data Acquisition

The variables of interest included age, sex, race, body mass index (BMI), comorbid medical conditions, admission, hospital course, and outcome characteristics. Admission characteristics included laboratory data collected within 48 h of admission, such as inflammatory markers; other hospital course characteristics such as treatments received and measures of organ dysfunction; and outcome data via medical record abstraction.

Admission characteristics included SpO_2_; administered FiO_2_; inflammatory laboratory markers, including C-reactive protein (CRP), lactate dehydrogenase (LDH), ferritin, and creatinine phosphokinase (CPK); and other laboratory markers, including creatinine, absolute lymphocyte count, D-dimer, troponin, leukocyte count, activated prothrombin time (aPTT), prothrombin time (PT), and international normalized ratio (INR). Admission chest radiographic findings were classified as normal, focal opacities, multifocal opacities, nodular lesions, or cavitations based on radiologic reports.

Hospital course characteristics included blood and lower-respiratory cultures if available during admission; treatments received, including the use of anticoagulation; and laboratory measures of organ dysfunction at the time of *Aspergillus* detection for each patient throughout admission for kidney injury (creatinine), cardiac injury (troponin), hepatic injury (ALT and AST), or coagulopathy (INR).

Outcome variables were defined as follows: (1) intensive care unit (ICU) admission; (2) length of ICU stay; (3) length of intubation; (4) readmission within 30 days; (5) 30-day and 60-day inpatient outcomes defined as discharged, remained inpatient, death, or hospice/DNI; and (6) the need for home oxygen support.

### 2.5. Statistical Analysis

Patient characteristics are reported as either total numbers and percentages when grouped or as medians and standard deviations when numeric. Laboratory values are reported as medians and standard deviations to limit the impact of outlier values. Differences between groups were evaluated with Welch’s *t*-test or Fisher’s exact test when group sizes were small. Associations between characteristics and outcomes (e.g., risk of invasive aspergillosis, mortality) were estimated with univariable logistic regression, reported as odds ratios with 95% confidence intervals. Confidence intervals for incidence and mortality were estimated using the Clopper–Pearson interval. Associations between characteristics and outcomes were estimated with multivariable logistic regression, reported as odds ratios with 95% confidence intervals. Characteristics with a small sample size (<4) were excluded, as these generated unstable estimates in the multivariable models.

To estimate the mortality rate and incidence of IPA in patients with COVID-19, we obtained all incident COVID-19 cases at our main facilities and excluded patients not eligible for our COVID-19/IPA cohort due to age or hospitalization status. We included incident patients in our cohort of patients with COVID-19 and IPA. Mortality data were most recently updated on 20 August 2021. All analyses were performed in R version 3.6.3 and RStudio version 1.4.1717 using the packages dplyr, tidyverse, and forestplot [26,27,28,29,30].

## 3. Results

During the observation period from March 2020 and May 2021, 2461 patients were admitted with a diagnosis of a SARS-CoV-2 infection. Of these, 459 (18.7%) required ICU admission. Lower-respiratory-tract samples were positive for *Aspergillus* in 30 patients with COVID-19. Twenty-one patients (70%) were classified as probable IPA, and nine patients (30%) were classified as *Aspergillus* colonization. No biopsies were obtained in any of the patients, precluding the ability to classify any patients as definite IPA. The prevalence of *Aspergillus* isolated in hospitalized COVID-19 patients in our institution was 1.18%. CAPA prevalence was 0.85% (0.53–1.3) among these COVID-19 hospitalized patients and 4.6% (2.9–6.9) in critically ill ICU patients with COVID-19.

The characteristics of patients with *Aspergillus*-positive lower-respiratory-tract samples are detailed in Table 1. Most of these patients were Black (53.3%), male (63.3%), and older, with a median age of 66 and a standard deviation of 11.6 years. Of the patients, 73.3% were over the age of 65 years. The median BMI was 28.05 kg/m^2^. Comorbidities were highly prevalent among these individuals: 67% had a history of hypertension, 40% a history of diabetes, 37% a history of chronic lung disease, 33% a history of heart disease, and 20% a history of chronic kidney disease. Two patients were immunocompromised (6.7%). Among the 30 patients identified as having CAPA in our study, at the time of diagnosis, CXR or thorax CT revealed: focal or lobar opacities for 8 patients, diffuse or multifocal opacities for 19 patients, nodular lesions for 2 patients, and cavitations for 2 patients. The time between COVID-19 and aspergillus diagnoses ranged between −3 (IPA diagnosed prior to COVID-19) and 391 days, with a median of 14 days. Regarding COVID-19 treatment, 7 patients received hydroxychloroquine (23%), 10 patients (33.3%) received remdesivir, and 4 patients received tocilizumab (13.3%), as established by the local protocol for severe COVID-19 pneumonia.

We compared the characteristics of the probable CAPA and colonization groups in Table 1. There were no significant differences between the two groups including demographics, risk factors, or clinical parameters. There were also no significant differences between the two groups regarding the COVID-19 treatment received. All patients were positive for *Aspergillus* species in their respiratory-tract cultures. *Aspergillus fumigatus* was the most common species causing co-infection in COVID-19 patients (76.7%), followed by *Aspergillus flavus* (16.7%). The following species were isolated; 23 *A. fumigatus*, 5 *A. flavus*, 4 *A. niger*, and 2 *A. terres*. Concomitant bacterial infections were found in 10 patients (33.3%). Twenty-eight patients (93.3%) received antimicrobials, and all patients received corticosteroids (≥20 mg/day of prednisone equivalent). Antifungal therapy with voriconazole or isavuconazole was initiated in 15 of the 21 (71%) patients with CAPA. Patients with CAPA were more likely to be treated with antifungal treatment, 62% vs. 22%; however, this difference was not statistically significant.

Twenty-seven patients (90%) with COVID-19 and *Aspergillus* detected in their respiratory culture were on mechanical ventilation. The median total length of ICU stay was 14 days ± 11.6, and the median duration of ICU stay prior to the isolation of the first *Aspergillus* spp. sample was 4 days. Fifteen patients (50%) required continuous renal replacement therapy during their hospital stay. All 30 patients required oxygen therapy, with an average PaO_2_/FiO_2_ ratio of 68.88 indicative of severe ARDS.

**Figure 1:**
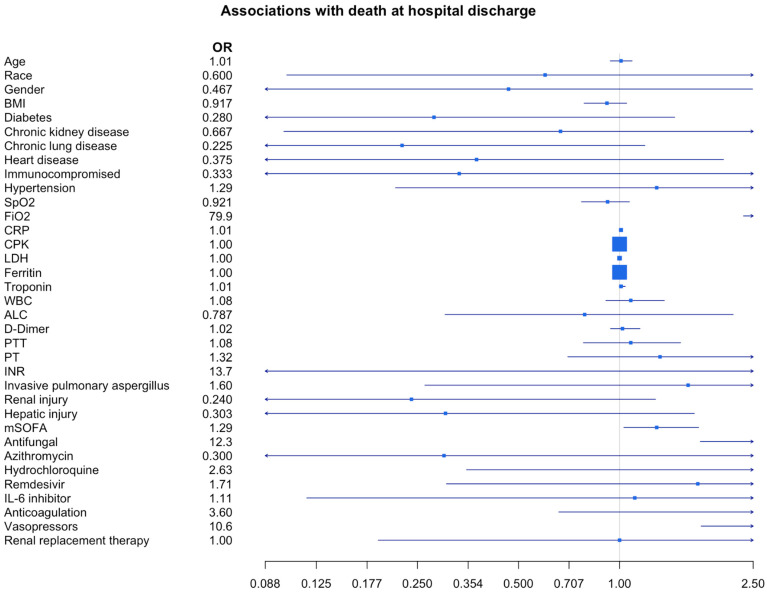
Associations with death at hospital discharge. Forest plot of mortality by demographics, risk factors, and clinical parameters of patients with COVID-19 and aspergillus (colonization or invasive). Associations between characteristics and mortality were estimated with univariable logistic regression, reported as odds ratios with 95% confidence intervals. Confidence intervals for incidence and mortality were estimated using the Clopper–Pearson interval.

In the analysis of hospital patients at discharge, eight patients were discharged alive. In-hospital mortality was 73%. In-hospital mortality between CAPA and COVID-19 patients with *Aspergillus* colonization was not significantly different (76% vs. 67%, *p* = 1.00; OR 1.60, 0.263–8.87, *p*-value = 0.590). The overall in-hospital mortality for COVID-19 patients in whom *Aspergillus* was not isolated was 15.8%, and it was 64.5% for those who required admission to the ICU. There was no significant difference in mortality between critically ill patients with *Aspergillus* compared to those without (73.3% vs. 64.5%, OR 1.53, CI 0.64–4.06, *p* = 0.43). The variables that were significantly associated with in-hospital mortality in patients in whom *Aspergillus* was isolated according to univariate analysis were the use of antifungal therapy (*p* = 0.035), vasopressors (0.016), and a higher mSOFA score (*p* = 0.043, OR 1.29 CI 1.03–1.72) (Table 2, Figure 1). In the multivariable model adjusting for over-significant variables, FiO_2_ was the only variable associated with in-hospital mortality in patients in whom *Aspergillus* was isolated (OR 1.07, 95% CI 1.01–1.27) (Supplemental Table S1).

## 4. Discussion

This is one of the largest studies on CAPA conducted in the US and included a predominantly African American population. Many case reports and retrospective studies of IPA in COPD patients and non-immunocompromised patients with COVID-19 have been published [18,20,24,31,32]. We reported probable CAPA in 21 of 30 COVID-19 patients with *Aspergillus* species isolated from the lower respiratory tract and without underlying immunocompromising diseases. A strength of our study was the use of lower-respiratory-tract samples obtained via tracheal suctioning and bronchoalveolar lavage to identify probable invasive aspergillus cases, which were more likely to accurately delineate invasive pulmonary infection from colonization than upper-respiratory-tract samples given their proximity to the infection.

Our study of a large number of hospitalized COVID-19 patients showed that *Aspergillus* was detected in 0.85% of patients and 4.6% of ICU patients. Furthermore, the study showed that 70% of these patients fulfilled the criteria for probable CAPA, while the rest were classified as colonization. The prevalence of CAPA in our patients was on the lower end of the range reported in the literature, which is 3–33% [6,33].

The mortality of those with *Aspergillus* isolated from lower-respiratory-tract samples was similar to that of patients with CAPA vs. colonization (76.2% vs. 66.7%, OR 1.60, 0.263–8.87, *p* = 0.590). There was no significant difference in the hospital outcomes between all ICU COVID-19 patients and ICU COVID-19 patients in whom *Aspergillus* was detected (OR 1.53, CI 0.64–4.06, *p* = 0.43).

In over a year of the COVID-19 pandemic at our institution, 2461 adult patients with laboratory-confirmed COVID-19 were admitted. Of these patients, 459 (18.7%) required ICU admission. CAPA was diagnosed in 0.85% of the 2461 patients with COVID-19 hospitalized within our institution. Almost all patients with CAPA and *Aspergillus* colonization were intubated and received a course of corticosteroids. No lung biopsies or autopsy samples were obtained in our COVID-19 patient population, likely due to concern for personnel safety.

Initial research on risk factors for *Aspergillus* infection and the treatment of invasive disease focused primarily on immunocompromised patients. However, a recent review of the literature indicated an expanding spectrum of patients at risk for IPA. Patients with ARDS secondary to influenza are prone to IPA in the absence of any immunodeficiency [17,25,34]. Other underlying comorbidities such as COPD, diabetes mellitus, cirrhosis, chronic alcohol use, and HIV are associated with IPA [11]. Our study showed similar underlying comorbidities: diabetes (40%), chronic lung disease (36.7%), heart disease (33.3%), and hypertension (66.7%). Additionally, an abnormal BMI was noted in 73% of our patients (overweight: 33.3%, obese: 33.3%, and BMI >40: 6.7%). Similar to previous studies, COVID-19-induced ARDS was also accepted as a host risk factor in our study [10,18]. Some studies have suggested the role of immunomodulating drugs in developing CAPA, specifically in studies of patients with severe influenza [24,25,35]. Most of our patients received corticosteroids and some received tocilizumab, which may be a potential risk factor for CAPA [36].

### 4.1. Mortality in Different Diagnostic Categories

The in-hospital mortality of patients with CAPA in this study was high (76.2%) but consistent with prior reports of mortality rates [37]. In the 30 patients with COVID-19 pneumonia with any *Aspergillus*-positive laboratory finding, there was a 73.3% (54.1–87.7) mortality rate. Mortality observed among cases with probable IPA (76.2%) and *Aspergillus* colonization (67%) was not significantly different (*p* = 1.00). In comparison, the in-hospital mortality rate of COVID-19 patients was only 15.8% (14.9–17.3) within the same duration, and it was 64.5% (59.9–68.9) among COVID-19 patients in the ICU setting. Interestingly, the patients treated with antifungal therapy (63.6% vs. 12.5%, respectively; *p* = 0.035) had significantly higher rates of mortality. For patients treated with antifungal therapy, the mortality rate was 99.3% (68.1–99.8). However, this could be attributed to the fact that patients with a worse overall disease severity were more likely to be treated with antifungal therapy. In our multivariable model, only lower FiO_2_ was significantly associated with increased mortality in CAPA or colonized populations. The mortality rate of 73.3% in our study is concerning: regardless of the classification of colonized or probable, CAPA carries a high mortality rate. Our study is consistent with other studies showing mortality rates greater than 50% [10,18,38,39,40,41,42].

Our study suggests an increased risk for critically ill COVID-19 patients of developing co-infection with *Aspergillus,* which has been linked to increased mortality rates. Therefore, testing for the presence of *Aspergillus* in respiratory secretions and galactomannan in serum samples in critically ill COVID-19 patients should be considered in order to identify patients who may require intensive treatment.

### 4.2. Limitations

Our study had several limitations, as it was a retrospective study at a single center. There were no predefined CAPA screening, fungal diagnostics, or treatment protocols at our institution. There was also an absence of tissue-proven diagnosis, which accurately identifies invasive infection but is often too prohibitive to obtain in clinical contexts. We correspondingly categorized these cases as “probable invasive aspergillus” to reflect this uncertainty. Our study was also limited by the number of COVID-19 patients with *Aspergillus* isolated in the respiratory cultures, which limited the power of our analyses to identify differences in risk factors for invasive aspergillus and mortality; however, this is inherent for relatively rare conditions.

## 5. Conclusions

Identifying patients at higher risk for complications and mortality is crucial in cases of COVID-19 infection. Our results emphasized worse outcomes for COVID-19-associated IPA in critically ill patients despite appropriate treatment. Due to the high CAPA-related mortality, we support the prompt initiation of antifungal treatment in critically ill patients for whom respiratory *Aspergillus* spp. has been isolated and who fulfill the CAPA criteria. Future studies should evaluate whether antifungal prophylaxis may reduce CAPA prevalence.

A complicated issue in COVID patients with ARDS is differentiating *Aspergillus* colonization from IPA, especially since radiological imaging is non-specific. Therefore, testing for the presence of *Aspergillus* in lower-respiratory secretions and galactomannan in serum samples in critically ill patients with COVID-19 should be considered. Our findings highlight the need for further studies to address whether there is a potential association between aspergillosis and COVID-19 and if it is a relevant complication in critically ill patients with COVID-19 pneumonia. Larger prospective cohort studies are needed to identify prognostic factors to allow for more effective prevention.

## Figures and Tables

**Table 1:** Characteristics of CAPA patients vs. COVID-19 patients with Aspergillus colonization. Probable invasive aspergillus infection vs. aspergillus colonization by demographics, risk factors, and clinical parameters of patients with COVID-19. Patient characteristics are reported either as total numbers and percentages when grouped or as medians and standard deviations when numeric. Laboratory values are reported as medians and standard deviations to limit the impact of outlier values. Differences between groups were evaluated with Welch’s *t*-test or Fisher’s exact test when group sizes were small. Associations between characteristics and risk of invasive aspergillosis were estimated with univariable logistic regression, reported as odds ratios with 95% confidence intervals. Confidence intervals for incidence and mortality were estimated using the Clopper–Pearson interval.

Characteristics		Colonization (9)	Probable (21)	*p*-Value
Age group (*n*, %)				0.1302
	<40 years	2 (22%)	0	
	40–64 years	1 (11%)	5 (24%)	
	≥65 years	6 (67%)	16 (76%)	
Race				0.8718
	Black	5 (56%)	11 (52%)	
	White	2 (22%)	3 (14%)	
	Other	2 (22%)	7 (33%)	
Gender				0.1682
	Male	3 (33%)	16 (76%)	
	Female	6 (67%)	5 (24%)	
Body mass index				1
	<18.5	0	1 (5%)	
	18.5–24.9	2 (22%)	5 (24%)	
	25–29.9	3 (33%)	7 (33%)	
	30–39.9	3 (33%)	7 (33%)	
	>40	1 (11%)	1 (5%)	
Medical history				
	Diabetes	5 (56%)	7 (33%)	0.4181
	Chronic kidney disease	2 (22%)	4 (19%)	1
	Chronic lung disease	4 (44%)	7 (33%)	0.6871
	On home oxygen	1 (11%)	1 (5%)	0.5172
	Heart disease	2 (22%)	8 (38%)	0.6749
	Liver disease	1 (11%)	1 (5%)	0.5172
	Immunocompromised	0	2 (10%)	1
	Neurologic disorders	1 (11%)	3 (14%)	1
	Hypertension	7 (78%)	13 (62%)	0.6749
	Hematologic malignancy with HSCT	0	1 (5%)	1
	Solid tumor w/o chemo	0	1 (5%)	1
	Long-term steroid use	1 (11%)	0	0.267
**Admission characteristics**				
Oxygen therapy (median ± SD)				
	SpO_2_	89 ± 5.61	92 ± 5.92	0.671
	FiO_2_	0.32 ± 0.240	0.5 ± 0.351	0.06498
Labs				
	CRP	81.8 ± 96.5	142 ± 77.9	0.2425
	CPK	598 ± 1041	146 ± 252	0.0795
	LDH	514 ± 608	482 ± 284	0.7725
	Ferritin	481 ± 1169	1186 ± 1301	0.3042
	Troponin	36 ± 94.5	42.5 ± 79.0	0.8469
	WBC	10.2 ± 4.60	6 ± 5.12	0.2305
	ALC	0.9 ± 1.27	0.85 ± 0.608	0.4987
	D-dimer	2.22 ± 12.8	1.80 ± 9.49	0.6716
	PTT	27.7 ± 3.56	30.5 ± 3.41	0.2061
	PT	12 ± 0.844	11.2 ± 2.33	0.8181
	INR	1.13 ± 0.0754	1.06 ± 0.234	0.8094
**Hospital course**				
Organ dysfunction				
	Renal injury	5 (56%)	13 (62%)	1
	Hepatic injury	4 (44%)	9 (43%)	1
	Cardiac injury	2 (22%)	2 (10%)	1
	Coagulation dysfunction	1 (11%)	2 (10%)	1
mSOFA				0.06276
	<8	1 (11%)	11 (52%)	
	8–11	2 (22%)	4 (19%)	
	>11	6 (67%)	6 (29%)	
Treatment				
	Antifungal	2 (22%)	13 (62%)	0.1086
	Azithromycin	0	4 (19%)	0.2874
	Hydroxychloroquine	2 (22%)	5 (24%)	1
	Other antibiotics	8 (89%)	20 (95%)	0.5172
	Remdesivir	3 (33%)	7 (33%)	1
	IL-6 inhibitor	0	4 (19%)	0.2874
	Anticoagulation	3 (33%)	11 (52%)	0.4397
	Vasopressors	8 (89%)	14 (67%)	0.3742
	Renal replacement therapy	6 (67%)	9 (43%)	0.427
Alive/dead				0.6662
	Alive	3 (33%)	5 (24%)	
	Dead	6 (67%)	16 (76%)	

**Table 2:** Comparison of characteristics by status at discharge. Mortality by demographics, risk factors, and clinical parameters of patients with COVID-19 and aspergillus (colonization or invasive). Patient characteristics are reported either as total numbers and percentages when grouped or as medians and standard deviations when numeric. Laboratory values are reported as medians and standard deviations to limit the impact of outlier values. Differences between groups were evaluated with Welch’s *t*-test or Fisher’s exact test when group sizes were small. Associations between characteristics and mortality were estimated with univariable logistic regression, reported as odds ratios with 95% confidence intervals. Confidence intervals for incidence and mortality were estimated using the Clopper–Pearson interval.

Characteristics		Alive (8)	Deceased (22)	*p*-Value
Age group (n, %)				0.256
	<40	1 (12.5%)	1 (4.5%)	
	40–64	0	6 (27.3%)	
	≥65	7 (87.5%)	15 (68.2%)	
Race				1
	Black	5 (62.5%)	11 (50%)	
	White	1 (12.5%)	4 (18.2%)	
	Other	2 (25%)	7 (31.8%)	
Gender				0.479
	Male	4 (50%)	15 (68.2%)	
	Female	4 (50%)	7 (31.8%)	
Body mass index				0.518
	<18.5	0	1 (4.5%)	
	18.5–24.9	2 (25%)	5 (22.7%)	
	25–29.9	1 (12.5%)	9 (40.9%)	
	30–39.9	4 (50%)	6 (27.3%)	
	>40	1 (12.5%)	1 (4.5%)	
Medical history				
	Diabetes	5 (62.5%)	7 (31.8%)	0.210
	Chronic kidney disease	2 (25%)	4 (18.2%)	0.645
	Chronic lung disease	5 (62.5%)	6 (27.3%)	0.104
	On home oxygen	0	2 (9.1%)	1
	Heart disease	4 (50%)	6 (27.3%)	0.384
	Liver disease	0	2 (9.1%)	1
	Immunocompromised	1 (12.5%)	1 (4.5%)	0.469
	Neurologic disorders	0	4 (18.2%)	0.550
	Hypertension	5 (62.5%)	15 (68.2%)	1
	Hx of malignancy (hematologic or solid)	0	2 (9.0%)	1
	Long-term steroid use	1 (12.5%)	0	0.267
**Admission characteristics**				
Oxygen therapy (median ± SD)				
	SpO_2_	91.5 ± 4.36	90.5 ± 6.15	0.256
	FiO_2_	0.36 ± 0.094	0.65 ± 0.35	<0.001
Labs				
	CRP	110 ± 70.2	124 ± 88.8	0.157
	CPK	213 ± 585	323 ± 789	0.586
	LDH	442 ± 185	582 ± 470	0.055
	Ferritin	714 ± 1188	1077 ± 1323	0.706
	Troponin	40 ± 27.8	36 ± 98.5	0.095
	WBC	6.20 ± 3.99	7.15 ± 5.37	0.383
	ALC	0.85 ± 1.26	0.9 ± 0.68	0.706
	D-dimer	0.97 ± 12.2	3.08 ± 9.62	0.713
	PTT	29.1 ± 2.92	29.5 ± 3.74	0.611
	PT	11.3 ± 0.618	11.3 ± 2.25	0.322
	INR	1.08 ± 0.066	1.06 ± 0.225	0.340
**Hospital course**				
Invasive pulmonary aspergillosis				0.666
	Probable	5 (62.5%)	16 (72.7%)	
	Colonization	3 (37.5%)	6 (27.3%)	
Organ dysfunction				
	Renal injury	3 (37.5%)	15 (68.2%)	0.198
	Hepatic injury	2 (25%)	11 (50%)	0.238
	Cardiac injury	0	4 (18.2%)	0.119
	Coagulation dysfunction	0	3 (13.6%)	1
mSOFA				0.054
	<8	6 (75%)	6 (27.3%)	
	8–11	0	6 (27.3%)	
	>11	2 (25%)	10 (45.5%)	
Treatment				
	Antifungal	1 (12.5%)	14 (63.6%)	0.035
	Azithromycin	2 (25%)	2 (9.1%)	0.284
	Hydroxychloroquine	1 (12.5%)	6 (27.3%)	0.638
	Other antibiotics	8 (100%)	20 (90.9%)	1
	Remdesivir	2 (25%)	8 (36.4%)	0.682
	IL-6 inhibitor	1 (12.5%)	3 (13.6%)	1
	Anticoagulation	2 (25%)	12 (54.5%)	0.226
	Vasopressors	3 (37.5%)	19 (86.4%)	0.016
	Renal replacement therapy	4 (50%)	11 (50%)	1

## Data Availability

Data are not available due to privacy restrictions.

## Supplementary Materials

The following supporting information can be downloaded at: https://www.mdpi.com/article/10.3390/arm91050027/s1, Supplemental Figure S1: Schematic flow chart depicting study design, Supplemental Table S1: Estimates from multivariable model for mortality risk. Mortality risk for characteristics after adjusting for all significant patient characteristics. Mortality risks were estimated with multivariable logistic regression, reported as odds ratios with 95% confidence intervals. Characteristics with a small sample size (<4) were excluded, as these generated unstable estimates in the multivariable models.
