# Supplementary material for: The Clinical Significance of Aspergillus Detected in Lower-Respiratory-Tract Samples of Critically Ill COVID-19-Positive Patients
Source: Adv Respir Med. 2023 Sep 2;91(5):337–49. doi: 10.3390/arm91050027 (PMC10514834; doi:10.3390/arm91050027)
Supplement: Supplementary file 1 [file arm-91-00027-s001.zip › arm-2398884-supplementary.pdf]

Supplemental Figure S1: Schematic Flow Chart Depicting Study Design

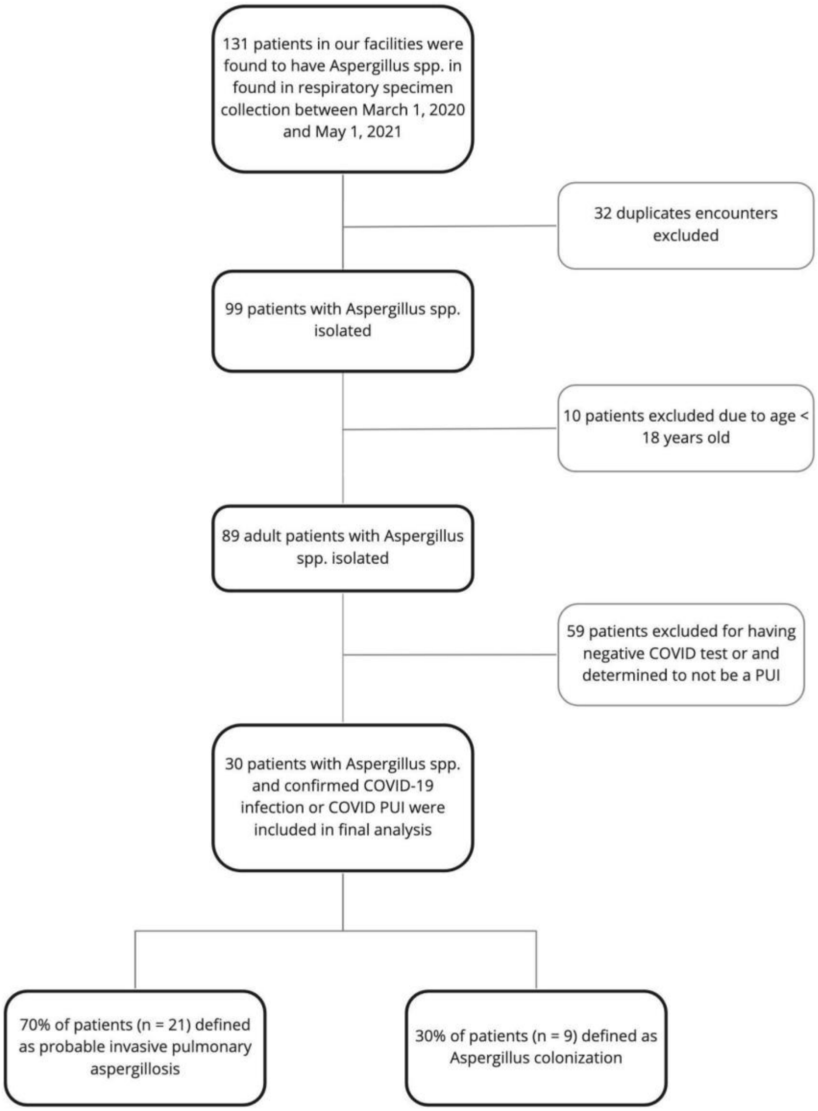

Supplemental Table S1. Estimates from multivariable model for mortality risk

Mortality risk for characteristics after adjusting for all significant patient characteristics. Mortality risks were estimated with multivariable logistic regression, reported as odds ratios with 95% confidence intervals. Characteristics with small sample size (<4) were excluded as these generated unstable estimates in the multivariable models.

Variable	OR	95% Interval
**Age**	1.05	0.880 – 1.45
**RRT**	1.42	0.0442 – 71.6
**FiO2 (%)**	1.07	1.01 – 1.27
**LDH**	1.00	0.990 – 1.01
**Trop**	1.02	0.982 – 1.07
